# Effects of a peer-led Walking In ScHools intervention (the WISH study) on physical activity levels of adolescent girls: a cluster randomised pilot study

**DOI:** 10.1186/s13063-017-2415-4

**Published:** 2018-01-11

**Authors:** Angela Carlin, Marie H. Murphy, Alan Nevill, Alison M. Gallagher

**Affiliations:** 1 Nutrition Innovation Centre for Food and Health (NICHE), Biomedical Sciences Research Institute, University of Ulster, Coleraine Campus, Coleraine, UK; 2 Centre for Physical Activity and Health, Sports and Exercise Sciences Research Institute, University of Ulster, Jordanstown Campus, Newtownabbey, UK; 3 School of Sport, Performing Arts and Leisure, University of Wolverhampton, Wolverhampton, UK

**Keywords:** Physical activity, Adolescent girls, Walking, Peer-led, School-based intervention

## Abstract

**Background:**

School-based interventions may be effective at increasing levels of physical activity (PA) among adolescents; however, there is a paucity of evidence on whether walking can be successfully promoted to increase PA in this age group. This pilot study aimed to assess the effects of a 12-week school-based peer-led brisk walking programme on levels of school-time PA post intervention.

**Methods:**

Female participants, aged 11–13 years, were recruited from six post-primary schools in Northern Ireland. Participants were randomized by school (cluster) to participate in regular 10–15-min peer-led brisk walks throughout the school week (the WISH study) (*n* = 101, two schools) or to continue with their usual PA (*n* = 98, four schools). The primary outcome measure was school-time PA post intervention (week 12), assessed objectively using an Actigraph accelerometer. Secondary outcome measures included anthropometry, cardiorespiratory fitness and psychosocial measures. Changes in PA data between baseline (T0) and end of intervention (week 12) (T1) were analysed using a mixed between-within subjects analysis of variance with one between (group) and one within (time) subjects factor, with two levels.

**Results:**

Of 199 participants recruited (mean age = 12.4 ± 0.6 years, 27% overweight/obese), 187 had valid accelerometer data for inclusion in subsequent analysis. A significant interaction effect was observed for changes in light intensity PA across the school day (*p* = 0.003), with those in the intervention increasing their light intensity PA by 8.27 mins/day compared with a decrease of 2.14 mins/day in the control group. No significant interactions were observed for the other PA measures across the intervention. Intervention effects on school-time PA were not sustained four months post intervention.

**Conclusions:**

The intervention increased daily light intensity PA behaviour in these adolescent girls but did not change moderate to vigorous physical activity (MVPA). These findings suggest that a school-based brisk walking intervention may be feasible and can change PA behaviour in the short term, but it is possible that the self-selected walking speeds determined by a peer-leader may not be sufficient to reach MVPA in this age group. Further research is needed to evaluate the potential of school-based brisk walking to contribute to MVPA in adolescent girls.

**Trial registration:**

ClinicalTrials.gov, NCT02871830. Registered on 16 August 2016)

## Background

Physical inactivity is a global health problem and is the fourth leading risk factor for mortality, contributing to approximately 3.2 million deaths per year worldwide [1]. Within the UK, approximately half of children aged seven years are failing to meet the current physical activity (PA) guidelines of at least 60 min of moderate to vigorous physical activity (MVPA) per day [2]. Children from Northern Ireland were the least likely to meet the recommendations, with 43.4% of children achieving 60 min per day of MVPA compared with the UK average of 51% [2]. The problem of physical inactivity appears more prevalent among girls, who consistently spend less time engaged in MVPA compared to boys [2–4]. Furthermore, girls also tend to participate in fewer sports compared with boys [5].

Given the health benefits of regular participation in PA on young people’s health [6, 7], the promotion of PA behaviours during youth is suggested as an important method for halting the decline in PA that occurs in the transition to adulthood. Participation in PA during adolescence can be an important contributor to levels of PA in adulthood [7], with evidence highlighting that PA behaviours track reasonably well from childhood to adulthood [8]. Adolescence represents a period of transition, with the move from primary to secondary education shown to influence young people’s PA behaviour [9–11]. A number of studies have identified barriers to PA participation in this age group, with commonly cited factors including lack of time due to academic commitments [12, 13] and a desire to spend free time engaged in activities that are not physically active, for example, socialising [14–16]. Other commonly cited barriers include off-putting uniforms [15] and cost and access to resources [12, 16]. There is a strong need to develop interventions that address these barriers and provide opportunities for this group to be active with friends in an environment that is fun and informal [14].

The provision of extra-curricular PA within the school environment often reflects the content of the physical education (PE) curriculum, i.e. team-based, structured sports [17]. Provision of PA within schools which promotes activities likely to be sustained into adulthood may be more effective [17], with evidence suggesting that both the type of PA individuals engage in during adolescence and the duration of participation can have an influence on PA behaviours in adulthood [18]. Given the relatively small contribution such team-based, structured sports make to PA in adulthood as compared with walking and other activities [19], it is important that other forms of PA are promoted among children and adolescents.

Walking is the most natural form of PA, requiring no specialist skills to participate [20], and can provide a practical, inexpensive option for children and adolescents to meet current guidelines [20, 21]. Brisk walking, accumulated in short bouts can contribute to the MVPA guidelines in children [22]. To date, research on walking to increase PA in children and adolescents is limited [23]. Findings from studies evaluating the impact of active commuting on PA have been inconsistent; a walking school bus intervention produced significant differences in MVPA [24], while other interventions demonstrated little or no effect of active commuting on overall PA levels [25, 26].

Although the contribution of walking to and from school on total PA should not be overlooked, given that this is unlikely to result in 60 min of MVPA, it is important to consider other ways to increase PA across the school day. A 15-week school-based accumulated brisk walking intervention in children (aged 5–11 years) resulted in increased objectively measured mean daily PA levels during school hours [27]. However, there is a paucity of data on the effectiveness of school-based walking interventions to increase PA among adolescents [23]. Furthermore, there is limited objective evidence on the effectiveness of PA interventions delivered during school recess (break and lunchtime), particularly in adolescent populations [28]. The majority of recess-based interventions involved in the use of playground markings and equipment to promote PA [28], which may not be as suitable in the post-primary school environment.

Social support, including logistical support, encouragement and role modelling have all been positively associated with levels of PA participation in young people [29]. Peer modelling, through the use of fictional characters, has been shown to change dietary habits in children [30] and, more recently, PA [31]. However, despite the level of evidence highlighting the relationship between PA and social support and modelling, there is limited evidence on the use of real-life peer modelling to promote PA in adolescents [31].

The aim of this pilot study was to investigate the feasibility of peer-led brisk Walking In ScHools intervention (the WISH study) and to investigate the impact of participating in a 12-week school-based walking programme on school-time PA and sedentary behaviour post-intervention (week 12) and at follow-up (six months). The secondary aim was to examine the effects of the intervention on a range of health-related outcome measures.

## Methods

### Study design

WISH was a pilot study of a school-based clustered randomised controlled trial (RCT). Following completion of baseline measurements, participants were randomly allocated by school, using a computer-based random number generator to either receive the intervention or to act as controls. Each school was given an anonymous code and randomly allocated to one of two study arms until all six schools had been assigned a study arm. The procedure was performed by a researcher at the University, independent of the project. The study arms relating to each randomly allocated number were written down by the individual before randomisation and concealed in an envelope until all enrolled participants completed all baseline assessments and it was time to allocate the intervention. The individual responsible for randomisation then notified the research team. Given the nature of the intervention, blinding of schools and participants was not possible following randomisation. The researcher responsible for subsequent data collection and analysis was not blinded to group allocation. There were no variations to the methods after the trial had commenced.

### Eligibility and recruitment

A convenience sample of schools in Northern Ireland were invited to take part in the study. Following permission from school principals, invitational letters were sent to parents/ guardians of all female pupils aged 11–13 years attending six post-primary schools in Northern Ireland. Pupils were eligible to participate if they were free from any medical condition that would limit their participation in a brisk walking intervention.

Ethical approval was obtained from the University of Ulster Research Ethics Committee (REC/11/0236). Written informed consent was obtained from parents/guardians and written assent was obtained from adolescents before randomisation.

### Intervention

The WISH study was delivered over a 12-week period (March to June 2014) to participants attending schools randomly allocated to receive the intervention. Participants were provided with the opportunity to attend a number of structured 10–15-min walks spread across the school week before the first bell, at mid-morning break and at lunch time. These walks were led by older pupils (aged 15–17 years) trained as walk leaders. Walk leader training ensured that the leaders considered all possible safety concerns and emphasised the importance of the walks being performed at a brisk pace, i.e. at a pace sufficient to elicit moderate intensity PA in participants. To ensure that the walks were performed at a moderate intensity, walk leaders were taught to recognise the physiological indicators of moderate intensity, for example, feeling warmer, noting an increase in heart rate, breathing harder, yet still able to carry on a conversation. The content of the walk leader training was informed by a PA co-ordinator from a local Health and Social Care Trust. The training was facilitated by a member of the research team and delivered to walk leaders at a lunchtime session. All walk leaders were provided with a copy of the training manual.

Risk assessments were performed by a member of the research team alongside a member of school staff for each predetermined walking route. Two walk leaders facilitated each walk; one walking at the front of the group and one at the back to maintain the pace. Walk leaders were supported in their role through an online private social media group moderated by the researcher, where they were provided with advice and tips on facilitating the intervention.

The intervention content was developed using social cognitive theory (SCT) and exposed participants to a number of influences on self-efficacy [32], e.g. observing peer leaders and other participants taking part in the walking sessions exposed participants to vicarious experiences. Participants were provided with timetables of the planned group walks, detailing the start time and meeting location for each walk, and given weekly verbal reminders to attend the walking sessions from school staff and walk leaders. Participants were also provided with prompt cards from the research team containing general tips and advice in relation to brisk walking and information on setting goals. Schools were instructed to provide at least two walking sessions for participants to attend each day. Participants were initially instructed to attend at least three walking sessions per week (of 10–15 min in duration) and to increase the number of sessions that they attended to at least five walking sessions per week by week 12 of the intervention.

To ensure intervention fidelity, the researcher visited each intervention school on a fortnightly basis to monitor the duration and intensity of the peer-led walks and to ensure that the intervention was standardised across intervention schools. To encourage adherence to the intervention, each participant was provided with a ‘reward card’, which was stamped by a walk leader each time they completed a walk. Participants were able to accumulate stamps for each completed walk and once reward cards were completed (indicating attendance at six walking sessions), participants were entered into a draw to win small tokens, e.g. cinema vouchers, water bottles and stationery. Walk leaders were also required to keep a log of participants attending each walk. School staff assisted the walk leaders by monitoring attendance logs and ensuring the study protocol was adhered to.

During the intervention period, participants in the control group were instructed to continue with their normal PA habits. Following completion of the intervention, all control schools were provided with study resources to implement their own school-based brisk walking program. All study participants were provided with a certificate to acknowledge their participation in the study.

### Participant assessments

Measurement periods were standardised across all schools and were completed at three time points. All measurements were conducted within the classroom setting at each participating school. Recruitment and baseline measurements were undertaken between January and March 2014 (T0). The first follow-up measurements were taken during week 12 of the intervention (T1) and the second follow-up (T2) was conducted after six months, during the first term of the following school year (September – October 2014).

The primary outcome measure was school-time PA, assessed objectively using the Actigraph GT3 accelerometer (Actigraph LLC, Pensacola, FL, USA). Participants were instructed to wear the device during all waking hours for seven consecutive days (including weekends), removing it only for bathing, taking part in water-based activities, such as swimming, and when asleep. To encourage compliance to the accelerometer protocol, participants were provided with reminder sheets to place in a prominent area within the home and a diary to log wear time and record periods of non-wear. Distribution and collection of monitors was during face-to-face meetings between the researcher and participants in the school.

A sampling epoch of 15 s was employed during data collection. All data were downloaded and analysed using Actigraph software V.6.5.4 (Actigraph, Pensacola, FL, USA). Participants were included in analysis if they had ≥ 3 days of valid wear time (i.e. ≥ 600 min/day) [33], but it was not compulsory for a participant to have at least one valid weekend day of data at each time point to be included in the analysis. The Evenson Actigraph cut-points [34] were used to estimate time spent in sedentary behaviour and light, moderate and vigorous intensity PA.

A school-time filter was applied during data analysis to assess the impact of the intervention on school-time PA. The filter was applied to capture any PA during 08:30–16:00 on weekdays that participants were due to attend school (this wear time filter was based on an average timetabled school day). PA data were analysed for mean time spent in sedentary behaviour and light, moderate and vigorous intensity PA, and for total daily PA.

Height (cm) and weight (kg) were measured to the nearest 0.1 cm and 0.1 kg, respectively, using a freestanding stadiometer (Leicester Height Measure), and digital scales (Seca 877). Height and weight were used to calculate body mass index (BMI) (kg/m^2^), with cut-points used to define weight category [35]. Waist circumference (cm) was measured to the nearest 0.1 cm, using an anatomical measuring tape.

Cardiorespiratory fitness was assessed using the Queens College Step Test [36]. Participants wore a heart rate monitor (Polar series, Polar Electro Inc, Kempele, Finland) during the step test, with heart rate recorded at baseline, and at 10 s, 15 s and 20 s following completion of the step test. An average of these three post-test measurements was used to predict maximum oxygen uptake capacity [37], expressed as mL∙kg^−1^∙min^−1^.

Participants also completed a previously validated questionnaire designed to assess self-efficacy for PA [38]. The questionnaire was adapted to also assess self-efficacy for walking, for example, ‘I could exercise even if I was tired’ was adapted to ‘I could walk even if I was tired’. Social support for PA and walking from male and female parents/guardians as well as friends were assessed using a five-item Likert scale [39]. Participants also completed the perceived benefits and barriers to exercise scale [40].

### Sample size considerations

A prospective power calculation was carried out based on data from Lee et al. [41]. The number of participants required to show significance at a 95% confidence interval (mean difference 467 steps/day) was calculated to be 50 participants per group, totalling 100 participants. To account for potential 50% drop-out and loss of accelerometer data due to non-wear over the course of the 12-week intervention and longer-term four-month follow-up, the researchers aimed to recruit 100 participants to each study arm.

### Statistical analyses

Data analysis was conducted using SPSS for Windows (Version 22, SPSS Inc, Chicago, IL, USA). Data are expressed as mean ± SD throughout unless otherwise stated. Changes in PA data were analysed using a mixed between-within subjects analysis of variance, to assess the two main effects of time and group and the interaction between these. Changes in PA between baseline (T0) and end of intervention (week 12) (T1) were analysed using a mixed between-within subjects analysis of variance with one between factor (group) and one within factor, with two levels (time). Data across all three study time points (T0, T1, T2) were analysed using a mixed between-within subjects analysis of variance with one between (group) and one within, with three levels (time) subjects factor. Where significant differences were observed, *post hoc* tests were conducted with adjustment for multiple comparisons. Repeated measures ANOVAs were used for within-group comparisons across the three study time points. A *p* value of < 0.05 was considered statistically significant.

## Results

### Recruitment

Of the 17 schools initially invited to take part in the study, six schools agreed to participate; three schools declined to participate on the basis of time constraints and eight schools did not reply to the initial invitational letter. Invitational letters were sent to 600 parents/guardians in the six schools. A total of 199 parents/guardians and participants returned consent and assent forms (33% response rate). All 199 participants were eligible for inclusion and underwent baseline measurements. Time point 2 measurements were conducted towards the end of the summer term, with a number of participants absent for measurements, largely due to family holidays or being unable to wear the accelerometer due to finishing the school year early for academic trips.

### Characteristics of participants

**Fig. 1:**
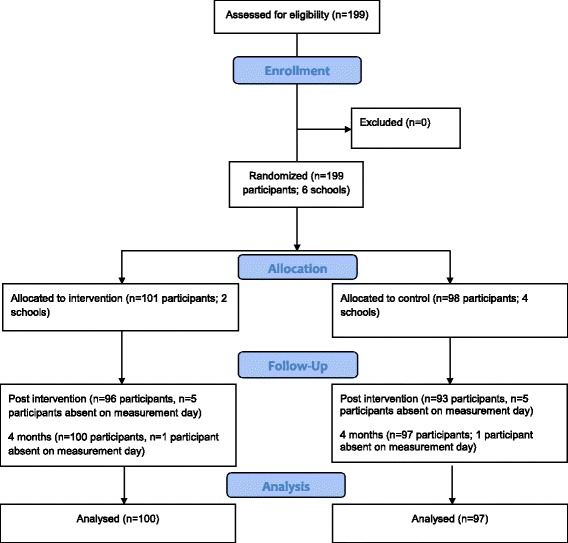
Flow of study participants

The flow of participants through the study is summarised in Fig. 1. One participant had no accelerometer data for time point 1 due to losing their accelerometer. One participant declined to have their weight measured at all three time points. A number of participants were unable to complete the step test at each time point due to injuries. Baseline (T0) characteristics of intervention (*n* = 101) and control (*n* = 98) participants are shown in Table 1.

**Table 1:** Baseline characteristics of WISH study participants

	Control (*n* = 98)		Intervention (*n* = 101)	
	Mean	SD	Mean	SD
Age (years)	12.16	0.51	12.54	0.57
Height (cm)	153.28	7.80	155.99	6.59
Weight (kg)	46.05	10.39	49.78	11.60
BMI (kg/m^2^)	19.25	4.27	20.31	3.86
BMI category [34] (% participants)				
Healthy weight	58.8		64.4	
Underweight	14.4		8.9	
Overweight	23.7		22.8	
Obese	3.1		4.0	
WC (cm)	62.64	8.51	65.20	8.70
Aerobic capacity (mL∙kg^−1^∙min^−1^)	34.57	3.48	34.78	7.73

*BMI* body mass index, *WC* waist circumference Cole cut-points [35]

### Physical activity

There were no differences in accelerometer wear time between groups for mean total minutes per day of wear (*p* = 0.633) or mean total days of valid wear (*p* = 0.804) at T0. However, significant differences for mean total wear time (min/week) were observed between intervention (3715.32 min/week) and control (3081.36 min/week) (*p* = 0.002). Significant differences were also observed for mean total days of valid wear between intervention (3.78 days/week) and control (4.61 days/week) groups at week 12 (T1) (*p* = 0.001).

When the wear time criteria were applied, there were no significant differences between those meeting the minimum wear time criteria and those not meeting the criteria in relation to height, weight, BMI or self-reported PA (all *p* > 0.05). At T0, 13% of participants were achieving the recommended 60 min of MVPA per day, with the majority of participants achieving 30–59 min per day of MVPA (57%), and the remaining 30% of participants failed to achieve at least 30 min per day of MVPA.

### Effect of intervention

The primary outcome measure was change in school-time PA at week 12 (T1); 117 participants (58.8% of total sample; *n* = 65 control, *n* = 52 intervention) had valid accelerometer data for T0 and T1 and were included in subsequent analysis (Table 2). There was a significant main effect of time observed for total daily school-time PA (*p* = 0.007), with both groups showing an increase in total daily PA. The overall interaction effect for group*time was non-significant. The main effect comparing both groups was significant, with those in the intervention group increasing total daily school-time PA by 9.2 min/day compared with an increase of 1.2 min/day in the control group (F(1,115) = 7.74, *p* = 0.007, partial eta squared (n^2^) = 0.061, moderate effect size). A main effect of time was observed for sedentary behaviour (*p* = 0.049), with a greater reduction in sedentary behaviour observed for the intervention group compared with the control group (F(1,115) = 6.40, *p* = 0.013, n^2^ = 0.053); however, the overall interaction between group and time was non-significant. A significant group*time interaction was observed for light intensity PA across the school day (F(1,115) = 9.30, *p* = 0.003, n^2^ = 0.075, moderate effect size), with a significant difference also observed between groups (increased by 8.3 min/day among intervention participants, compared with a decrease of 2.1 min/day among control participants) (F(1,115) = 5.80, *p* = 0.018, n^2^ = 0.048, small effect size). No significant time*group interactions were observed for time spent in moderate PA across the school day. A significant time*group interaction was observed for vigorous PA across the school day (*p* = 0.009); however, no differences were observed for time or between groups.

**Table 2:** Objectively measured habitual school-time^a^ physical activity and sedentary behaviour for intervention and control groups at baseline (T0) and week 12 (T1)

Time (min/day)	T0		T1			
	Mean	SD	Mean	SD	Change (95% CI)	*p* value^b^
Sedentary^c^						
Control^d^	310.30	24.85	308.31	38.22	−1.99 (−11.06–6.80)	0.013
Intervention^d^	325.13	21.80	316.31	24.28	−8.82 (−13.99– -3.64)	
Light PA^c^						
Control	118.56	20.32	116.42	21.83	−2.14 (−6.73–2.45)	0.018
Intervention	104.84	18.96	113.11	23.10	8.27 (3.22–13.32)	
Moderate PA^c^						
Control	16.80	6.39	19.84	7.91	3.05 (1.64–4.44)	0.122
Intervention	15.57	5.92	17.61	5.62	2.04 (0.52–3.57)	
Vigorous PA^c^						
Control	5.34	4.16	6.42	6.05	0.89 (−0.24–2.37)	0.071
Intervention	5.12	3.86	3.97	3.15	−1.15 (−2.03– -0.26)	
Total PA^c^						
Control	140.52	24.85	142.67	28.64	1.24 (−3.88–7.82)	0.007
Intervention	125.52	22.00	134.69	24.28	9.17 (3.92–14.41)	

*PA* Physical activity

^a^School-time filter (08:30–16:00)

^b^Differences between groups compared using mixed between-within subjects ANOVA

^c^Evenson cut-points [35]

^d^Participants with ≥ 3 days valid wear included in analysis (*n* = 65 control, *n* = 52 intervention)

Eighty-nine participants (44.7% of total sample; *n* = 48 control, *n* = 41 intervention) had valid PA wear time for inclusion in analysis of the longer-term effectiveness of the intervention (Table 3). There were significant differences between groups over time for minutes per day of sedentary behaviour, light and moderate intensity PA (Table 3). The positive effects observed at T1 for sedentary behaviour, light PA and total PA in intervention participants were not sustained at T2. Measurements taken four months post intervention highlighted sedentary behaviour had increased by 17 min/day among intervention participants compared with measurements at the end of the 12-week intervention, while light intensity PA decreased by 15.5 min/day.

**Table 3:** Objectively measured habitual school-time^a^ physical activity and sedentary behaviour for intervention and control groups at T0, T1 and T2

	Control (*n* = 48)		Intervention (*n* = 41)		Group time (*p*)^b^
	Mean	SD	Mean	SD	
Sedentary^c^					0.010
Baseline (T0)	309.94	24.81	325.08	18.90	
End of intervention (T1)^d^	310.41	38.11	317.12^e^	22.96	
4mths post-intervention (T2)^f^	319.89^g,h^	26.46	334.92^g,h^	20.90	
Light PA^c^					0.011
Baseline (T0)	118.73	19.88	105.18	16.83	
End of intervention (T1)^d^	117.94	20.09	112.47^e^	20.63	
4mths post-intervention (T2)^f^	104.08^g,h^	17.36	96.95^g,h^	18.07	
Moderate PA^c^					0.015
Baseline (T0)^i^	16.88	6.86	15.21	6.09	
End of intervention (T1)^d^	20.50^e^	8.26	17.41^e^	5.99	
4mths post-intervention (T2)	19.39^g^	7.11	14.82^h^	5.83	
Vigorous PA^c^					0.056
Baseline (T0)	5.44	4.39	5.09	3.96	
End of intervention (T1)	6.62	6.50	3.99	3.41	
4mths post-intervention (T2)	5.30	4.62	3.74^g^	2.79	
Total PA^c^					0.002
Baseline (T0)^i^	141.06	24.81	125.48	19.20	
End of intervention (T1)^d^	145.07	27.67	133.88^e^	22.96	
4mths post-intervention (T2)^f^	128.76^g,h^	22.44	115.50^g,h^	21.09	

*PA* Physical activity

^a^School-time filter (08:30–16:00)

^b^Differences between groups compared using mixed between-within subjects ANOVA

^c^Evenson [35]

^d^Significantly different (*p* < 0.05) changes between groups from T1 to T2

^e^Significantly different (*p* < 0.05) changes within groups from T0 to T1

^f^Significantly different (*p* < 0.05) changes between groups from T0 to T2

^g^Significantly different (*p* < 0.05) changes within groups from T0 to T2

^h^Significantly different (*p* < 0.05) within groups from T1 to T2

^i^Significantly different (*p* < 0.05) changes between groups from T0 to T1

### Secondary outcome measures

No significant changes were observed between T1 or T2 between groups for waist circumference (WC), WHR, BMI, cardiorespiratory fitness or blood pressure. A significant main effect was observed between groups for social support from friends across the study period (Table 4). Post hoc comparisons indicated that the mean difference between groups at T1 was significant, with a greater increase observed in the intervention group compared with controls. A significant difference was also observed between groups at T2. No other differences were observed for other sources of social support, for self-efficacy for walking and physical, or for perceived benefits and barriers to PA between groups.

**Table 4:** Scores for social support, self-efficacy and perceived benefits and barriers to exercise at T0, T1 and T2

	Control (*n* = 87)		Intervention (*n* = 96)		Group time (*p*)^a^
	Mean	SD	Mean	SD	
Social support from female parent/guardian					0.964
Baseline (T0)	3.14	0.70	3.11	0.71	
End of intervention (T1)	3.19	1.11	3.17	0.76	
4 months post intervention (T2)	2.99^b^	0.76	3.01	0.83	
Social support from male parent/guardian					0.220
Baseline (T0)	2.93	0.80	2.95	0.89	
End of intervention (T1)	2.80	0.83	3.02	0.87	
4 months post intervention (T2)	2.65^b^	0.83	2.83^c^	0.91	
Social support from friends					0.001
Baseline (T0)^d^	2.57	0.79	2.90	0.73	
End of intervention (T1)^e^	2.69	0.84	3.07^f^	0.72	
4 months post intervention (T2)	2.52	0.76	2.91	0.85	
Self-efficacy for PA					0.859
Baseline (T0)	1.85	0.47	1.94	0.52	
End of intervention (T1)	1.91	0.51	1.88	0.52	
4 months post intervention (T2)	1.98^b^	0.57	1.89	0.53	
Self-efficacy for walking					0.103
Baseline (T0)	1.85	0.48	1.78	0.51	
End of intervention (T1)	1.92	0.52	1.81	0.54	
4 months post intervention (T2)	1.95	0.59	1.81	0.55	
Perceived barriers to PA					0.340
Baseline (T0)	2.94	0.44	2.87	0.54	
End of intervention (T1)	2.99	0.56	2.89	0.52	
4 months post intervention (T2)	2.85^c^	0.55	2.83	0.66	
Perceived benefits to PA					0.177
Baseline (T0)	1.78	0.39	1.67	0.40	
End of intervention (T1)	1.77	0.45	1.71	0.45	
4 months post intervention (T2)	1.76	0.49	1.69	0.49	

*PA* Physical activity

^a^Differences between groups compared using mixed between-within subjects ANOVA

^b^Significantly different (*p* < 0.05) changes within groups from T0 to T2

^c^Significantly different (*p* < 0.05) within groups from T1 to T2

^d^Significantly different (*p* < 0.05) changes between groups from T0 to T1

^e^Significantly different (*p* < 0.05) changes between groups from T1 to T2

^f^Significantly different (*p* < 0.05) changes within groups from T0 to T1

## Discussion

This pilot feasibility study is the first peer-led structured school-based walking programme delivered in the post-primary school setting, aimed at investigating the impact of participating in a 12-week intervention on school-time PA and sedentary behaviour post intervention (week 12) and at follow-up (six months). The WISH study increased light intensity PA across the school day (08:30–16:00) with moderate effect size observed, highlighting the effectiveness of a 12-week school-based brisk walking intervention in eliciting changes in PA in this population. Light intensity PA increased by 8.3 min/day across the school day for the intervention group, compared with a decrease of 2.1 min/day within the control group. Based on these findings, a school-based walking programme may have the potential to increase light intensity PA by 45 min across the school week and subsequently reduce time spent in sedentary behaviours.

The increases observed in PA across the school day resulted from an increase in daily light intensity PA among intervention participants compared with controls. A significant interaction was not observed for sedentary behaviour within the present study, although findings were encouraging with those in the intervention group decreasing their levels of sedentary behaviour at a greater rate as compared with the control group. No differences were observed between groups for time spent in moderate or vigorous intensity PA at T1. To date, a limited number of walking interventions in children and adolescents have used accelerometers to measure PA outcomes [23], hampering comparisons with the present study. A moderate effect size was observed for changes in light intensity PA within the present study. Previous walking interventions in children have reported increases in MVPA ranging from 2.2 min/day [24] to 14 min/day MVPA [26]. The magnitude of school-based intervention effects has been shown to vary greatly across studies, with increases in MVPA in the range of approximately 5–45 min per week; however, differences in relation to total PA were not reported [42].

The WISH study increased levels of light intensity PA across the school day from baseline to 12 weeks. The decline in adolescent PA has been attributed to decreasing levels of light intensity PA as children move into adolescence [43]. A ten-year cohort study observed a decline in the contribution of light intensity PA to total overall PA from 19% at the age of five years to 8% at the age of 15 years [43]. Given that light intensity PA can contribute up to 30–40% of total daily PA, and that the activities that contribute to light intensity PA are likely to be more habitual in nature and less structured than moderate to vigorous activities [43], interventions that increase light intensity PA during the school day may be promising in efforts to halt the age-related decline in PA, particularly among adolescent girls who are at higher risk of being physically inactive [2, 4]. Furthermore, participation in light intensity PA may have beneficial associations with a number of cardio-metabolic biomarkers in adolescents [44].

The present intervention failed to increase levels of moderate intensity PA, with slight decreases observed in moderate and vigorous intensity PA at 12-week and six-month follow-up. Within the present study, the walk leaders were responsible for ensuring the walks were performed at a brisk pace, i.e. to elicit moderate intensity PA from participants. The findings from the present study suggest that the self-selected walking speeds of these peer leaders may not have been of sufficient pace to help adolescent girls achieve moderate intensity PA. Previous interventions provided heart rate monitors to participants to ensure walks were at least moderate intensity [27]. To ensure future interventions engage adolescents in levels of MVPA, heart rate monitoring or pedometers could be used to enable a real-time checking of exercise intensity. Previous walking studies targeted at adolescents have either failed to report outcomes in relation to walking intensity or have shown no changes in MVPA [45]; however, MVPA was measured using a self-report instrument within this study, making comparisons with the objective measures used within the present study difficult. Furthermore, the limitations involved in asking children to accurately recall exercise intensity should be noted, given their reduced ability to recall time and intensity compared with adults [46]. School-based walking interventions have been previously shown to increase objectively measured levels of MVPA in children, through the implementation of walking school buses [24, 26], highlighting that walking can be an effective means of increasing MVPA in youth. Increasing levels of MVPA in youth is of particular importance given the associated substantive health benefits produced by activity performed at moderate intensity [47].

The changes in light PA observed at 12 weeks were not maintained among intervention participants at four-month follow-up, highlighting that exposure to a 12-week walking programme was not sufficient to elicit longer-term behaviour change in this population. Although the WISH study provided adolescent girls with an activity that overcomes many of the issues associated with participating in competitive, team-based or vigorous activity in front of peers, increases in walking behaviour were not maintained once the intervention period ended. This may be attributable to a number of factors, e.g. the absence of walk leaders and/or structured walking sessions to attend post intervention. A lack of evidence on the longer-term effectiveness of PA interventions has been cited as a limitation of studies to date in children and adolescents [48]. A school-based structured walking intervention targeted at primary school children observed similar findings to the present study, within increases observed in mean daily PA [27] across the school day. The study did not include a longer-term follow-up; thus, it is unclear whether these increases were sustained. Given the differences that exist between primary and secondary education, for example, in relation to teaching provision and flexibility of the school day, it is difficult to draw comparison between walking interventions in children to the outcomes observed within the present study. To date, the follow-up times for other walking interventions targeted at adolescents have been in the range of 5–12 weeks (end of intervention measurements) [23] and not included longer-term follow-up. Consequently, there is a paucity of evidence on the longer-term effectiveness of walking interventions to increase PA in adolescents.

Unlike previous studies which have targeted walking behaviours on the commute to and from school, the WISH study aimed to provide extra opportunities for adolescent girls to be active at break and lunchtime. The limited number of intervention studies considering the impact of recess-based interventions makes it difficult to draw conclusions on the effectiveness of this approach compared with others, with no other structured walking interventions evidenced in adolescent girls [23]. Few studies to date have targeted PA during school recess and have mostly focused on the use of playground markings and games equipment to promote PA [28]. Given the lack of studies targeted at adolescents, it is difficult to draw conclusions on the suitability of such recess-based interventions on PA within this age group [28].

The present study highlights the feasibility of incorporating peer-led walks within the provision of a school’s extra-curricular PA. Peers can play a key role in influencing PA in children and youth, with evidence suggesting that young people model their PA behaviour on what other peers and friends are doing [49]. Developing peer support strategies, for example, encouraging strong friendships and having peers facilitate physical activities, may improve the outcomes of PA interventions targeted at inactive groups, e.g. adolescent girls [50]. Within the present study, older pupils volunteered to act as peer leaders and in turn, provided younger pupils with potential role models to encourage them to participate in increased PA. Peers have previously been identified as key role models among adolescent girls [51] and it is important that role models are incorporated within interventions targeted at this population [51]. The peer-led aspect of the present study targeted at an adolescent population is novel; evidence to date has shown the use of fictional peer modelling to be effective in change intake of fruit and vegetables [30] and more recently PA behaviours [31] in children. There is limited evidence on the use of peers to facilitate PA and how social groups within school can influence PA, owing in part to the focus on children’s studies within the literature, which tend to focus on interventions supervised and managed by teachers [52].

Within the WISH pilot study, increases in the mean score for social support from friends, but not from male or female parents/guardians, were observed for intervention participants, highlighting that the role of friends and other classmates may have been integral to participants’ engagement with the walking intervention. Previous research examining social support for PA in youth found that support from friends was the only source of support for lunchtime PA and was consistent across age groups [29]. Parents and friends have both been previously cited as key influences on current PA participation by adolescents [16], with parental support correlated with PA in children and adolescents [53, 54]. Parental encouragement has been previously significantly associated with lunchtime PA for junior school students only [29] but not for older pupils (aged 16–18 years) [29], suggesting that the influence of parents decreases as pupils move through adolescence and place more emphasis on the influence of peers [29]. Further research is needed to explore the role components of social support play in promoting PA behaviours among adolescent females [55] and future interventions should incorporate exposure to the multiple sources of social support within their design [55].

In the present study, participants in the intervention group wore the accelerometer for fewer days and fewer minutes per day than control participants. A number of approaches were employed to encourage adherence to wearing the accelerometer, for example, reminder phone calls, reminder leaflets to be placed in a prominent area of the home and provision of a diary for participants to log when they wore and removed the device [56]. While studies have reported that wearing accelerometers may be acceptable to most young people [57], a number have identified issues that may explain problems with compliance in this age group, e.g. adolescents may not view the monitor as ‘stylish’ [58]. Further strategies to improve compliance may include rewards for both wearing and returning the monitor or employing other technologies, e.g. smartphones to monitor PA behaviours.

### Strengths and weaknesses of the study

The WISH study is the first school-based brisk walking intervention targeted at adolescent girls. The pilot study was delivered in the normal school setting, which increases the ecological validity of the study, and was delivered by peers trained as walk leaders. The use of older peers to facilitate the intervention is novel. The intervention was low-cost and required few resources to implement within the school setting. PA-related outcomes were assessed objectively and a longer-term follow-up was included to assess the effectiveness of the WISH study once the intervention period had ended, which has been a limitation of previous studies in this population.

Although widely accepted, the use of accelerometers to measure PA in children and adolescents is not without its limitations; accelerometers cannot accurately measure certain activities, e.g. cycling and water-based activities [59], and wearing of accelerometers is not permitted in certain sports, for example, competitive team games or dance performances. Such limitations can lead to underestimations when using accelerometers to measure PA [59]. Furthermore, poor adherence to the accelerometer wear protocol was a limitation within the present study. Analysis of primary and secondary outcomes did not account for the clustering randomisation that took place at the school level; therefore, the results of this pilot study should be interpreted with caution. As this was a pilot study, further research is needed to explore the effectiveness of this intervention within a fully powered RCT.

## Conclusions

The WISH study demonstrated that levels of light intensity PA throughout the school day can be increased by participating in a brisk walking programme facilitated by older pupils trained as walk leaders. The positive intervention effects on light intensity PA are promising and may help offset the age-related declines in PA commonly observed among adolescent girls. Future research should examine the effects of walking during the school day on levels of MVPA in adolescent girls, which would help this population move towards meeting the PA guidelines. The findings of this study are of significant relevance to schools and policy-makers, indicating that the delivery of a low-cost walking intervention, requiring few resources to implement, is feasible within the school setting and indeed during the school day.

## Abbreviations

BMI: Body mass index
MVPA: Moderate to vigorous physical activity
PA: Physical activity
PE: Physical education
SCT: Social cognitive theory
WC: Waist circumference
